# How to do (or not to do) realist evaluations to advance theory, practice, and justice in health systems research

**DOI:** 10.1093/heapol/czaf080

**Published:** 2026-06-29

**Authors:** Praveenkumar Aivalli, Pragati Hebbar, Chinyere Mbachu, Prashanth Nuggehalli Srinivas, Seye Abimbola, Sara Dada

**Affiliations:** UCD School of Public Health, Physiotherapy and Sports Science, University College Dublin, Belfield, Dublin 4, Ireland; UCD Centre for Interdisciplinary Research Education and Innovation in Health Systems (UCD IRIS Centre), School of Nursing Midwifery and Health Systems, University College Dublin, Belfield, Dublin 4, Ireland; T. T. Narasimhan School of Advanced Studies at the Institute of Public Health Bengaluru, 3009, II-A Main, 17th Cross, Krishna Rajendra Rd, Banashankari Stage II, Bengaluru, Karnataka 560070, India; Health Policy Research Group, College of Medicine, University of Nigeria Nsukka, Old UNTH, Enugu 40001, Nigeria; Institute of Public Health, College of Medicine, University of Nigeria Nsukka, Enugu 40001, Nigeria; Department of Community Medicine, College of Medicine, University of Nigeria Nsukka, Enugu-Campus, Enugu 40001, Nigeria; Centre for Health Systems, Institute of Public Health, Bengaluru, India; School of Public Health, University of Sydney, Susan Wakil Health Building (D18), The University of Sydney, New South Wales 2006, Australia; Julius Global Health, University Medical Center, Utrecht University, P.O. Box 855003508 GA Utrecht, The Netherlands; UCD School of Public Health, Physiotherapy and Sports Science, University College Dublin, Belfield, Dublin 4, Ireland; UCD Centre for Interdisciplinary Research Education and Innovation in Health Systems (UCD IRIS Centre), School of Nursing Midwifery and Health Systems, University College Dublin, Belfield, Dublin 4, Ireland; School of Nursing, Midwifery and Health Systems, University College Dublin, Belfield, Dublin 4, Ireland

**Keywords:** health systems, realist, knowledge translation, policy, global health, context, power

## Abstract

The realist approach seeks to understand underlying mechanisms that explain how and why complex interventions, programmes, and policies work in specific contexts, making it particularly valuable in health policy and systems research (HPSR). We draw on reflexive practice of realist evaluations from several realist evaluation practitioners and on insights from an organized session at the Eighth Health Systems Research Symposium in Nagasaki, Japan, in 2024, where we engaged a diverse group of practitioners and researchers on how to use realist approaches in HPSR. Examples from our studies, while situated in distinct contexts, highlight common challenges in applying realist methodologies including identifying and refining context-mechanism-outcome configurations. Building on these examples, we illustrate how realist evaluations, if conducted rigorously and with the purpose of advancing justice in health systems, could do so through exposing structural barriers to health justice, amplifying local voices and fostering epistemic justice in knowledge production.

Key messages

1. Realist evaluation enables context-sensitive explanations of how and why health interventions succeed or fail, offering a valuable methodological lens to understand complexity and drive equitable reforms in health systems.
2. When approached reflexively, realist evaluation challenges dominant knowledge hierarchies by elevating the experiences and interpretations of marginalized actors, thereby fostering epistemic justice in health policy and systems research.
3. Undertaking realist evaluations in low- and middle-income countries often benefits from adaptive, context-aware strategies that can navigate institutional constraints and asymmetrical power relations while ensuring methodological integrity and fostering inclusive engagement.
4. Translating realist findings into meaningful policy and practice may be strengthened by participatory and locally rooted dissemination strategies that promote shared ownership and support justice-oriented and sustainable systems change.

## Introduction

Health system interventions rarely function in linear, predictable ways. They are embedded within social, political, and institutional structures that shape how policies are implemented and how actors engage with one another (Gilson 2011, Adam and de Savigny 2012). The field of health policy and systems research (HPSR) embraces the complex causality inherent in social systems (such as health systems) and seeks to explore, describe, and explain how (and why) health programmes and policies unfold differently, contingent upon the specific contexts. Traditional evaluation methodologies often focus on effectiveness regardless of context, failing to account for these intricate social, political, and institutional factors (DeGroff and Cargo 2009, Sheikh et al. 2011, Adam et al. 2012, Adam and de Savigny 2012, Peters et al. 2013, Prashanth et al. 2013, Jones 2018, Roche et al. 2021).

In response to these limitations, realist evaluation—a theory-driven, explanatory approach—has gained prominence in HPSR (Marchal et al. 2012). Rooted in the work of Pawson and Tilley (1997a, 1997b), realist evaluation is designed to uncover underlying mechanisms and examine how context shapes outcomes. While Pawson and Tilley focused on methodological realism, they paid less attention to the critical dimension of realism that addresses social injustice. Bhaskar’s Dialectical Critical Realism provides this ontological grounding, linking realist evaluation to questions of emancipation and justice (Bhaskar 2008, Mutua et al. 2025). Acknowledging this foundation strengthens our argument that realist evaluation can both explain outcomes and confront structural inequities. Thus, explanation is the purpose of realist evaluation, and realist studies typically draw on rich qualitative insights, such as interviews, observations, and document analysis, and, where appropriate, quantitative data to develop and refine programme theories. This combination of methods helps evaluators construct and test programme theories that are sensitive to local context. Its emphasis on explanation over mere measurement or at other times deterministic judgements of effectiveness, makes it particularly well-suited for interrogating complex dynamics such as power, governance, and institutional behaviour within health systems (Marchal et al. 2012, Ogilvie et al. 2020). Over the past decade, realist evaluation has been increasingly applied to explore intersectoral collaboration, community engagement, and health policy implementation in a range of contexts and settings (Marchal et al. 2012, Jagosh et al. 2015, Bergeron et al. 2017, Adhikari et al. 2019, Irvine Fitzpatrick et al. 2021, Mbachu et al. 2022, Hogeling et al. 2024, Hebbar et al. 2025).

Conducting realist evaluations in any context entails navigating complex decision-making structures, asymmetrical power relations, and varying levels of stakeholder participation, all of which shape knowledge production. However, in low- and middle-income countries (LMICs), these challenges are often compounded by resource constraints, less formalized research governance, and greater vulnerability to exploitation. These factors can heighten the risk of epistemic injustice by marginalizing local knowledge and limiting the capacity of participants to influence research processes and outcomes. Researchers often need to navigate varied decision-making structures, asymmetric power relations, and diverse forms of research participation, all of which have significant implications for knowledge production (Abimbola et al. 2019, Gilmore 2019, Broesch et al. 2020, Bryant 2022, Niati 2024, Alexandra 2025).

The dominant paradigm of evidence-based policymaking, which often privileges positivist methods, tends to marginalize approaches like realist evaluation that centre context, complexity, and local agency (Pawson 2006, Greenhalgh and Russell 2009). These challenges raise fundamental questions about epistemic justice (Abimbola 2023, Abimbola et al. 2024)—whose knowledge or learning counts in shaping health policies, programmes, and interventions? Whose interpretation or sense-making counts in framing their evaluation? How can methodologies attend to the potential to disregard (as knowers and interpreters of knowledge) people who are marginalized by the power structures that govern research or govern where research is conducted? This critical reflection on epistemic justice featured prominently as the central theme of the closing plenary at the Health Systems Research Symposium (HSR) in 2024, where these concerns were discussed in-depth by panellists (Health Systems Global 2024).

Epistemic injustice, a concept coined by philosopher Miranda Fricker, distinguishes testimonial and hermeneutical forms of injustice to describe how social power dynamics can marginalize certain people as knowers and interpreters (Fricker 2007). Epistemic injustice—that is, injustice done to a person or group in their capacity as knowers or interpreters of knowledge—may manifest as the discounting, dismissal, or silencing of those people’s testimony and accounts (testimonial injustice) or as the disregard, denial, or sidestepping of their interpretations and sense-making (hermeneutical or interpretive injustice) of their own reality, for example, in relation to the policy, programme, or intervention that is being evaluated (Bhakuni and Abimbola 2021). These two forms—testimonial injustice (stemming from credibility deficit suffered by marginalized others) and interpretive injustice (stemming from interpretive marginalization of those marginalized others)—constitute epistemic injustice (Abimbola et al. 2024). Realist evaluation’s explanatory focus on how and why interventions work in context makes it well suited to examining entrenched power differentials and the experiences of marginalized groups. When evaluators deliberately engage those most affected and attend to issues of credibility and interpretation, realist designs can help surface and challenge the epistemic injustices described above. However, simply adopting a realist approach does not automatically ensure epistemic justice; achieving that aim depends on how programme theories are formulated, who participates in refining them, and how findings are communicated.

In this article, we demonstrate how realist evaluation can be conducted in ways that are mindful of epistemic injustice—demonstrating specific strategies at each stage of the evaluation cycle, from developing programme theories to interpreting data and translating findings. As realist methodologies gain prominence in HPSR, their cross-contextual application presents a valuable opportunity to address the marginalization of local knowers and to promote epistemic justice in both research practice and policy engagement. We illustrate this argument by drawing on two recent realist evaluations. The first study (Aivalli) investigated power dynamics in intersectoral collaboration within district- and block-level governance structures in India. It examined how sectors such as health, nutrition, social welfare, and local governance interact in the implementation of health and nutrition policies. The second study (Dada et al. 2025a, 2025b) examined communication within community engagement processes in Eastern Province, Zambia, focusing on how and why community volunteers efforts to promote health-seeking work and how they are shaped by relationships among health workers, local leaders, and community members.

Drawing on these two evaluations, this paper offers a reflective and practice-informed guide for researchers, policymakers, and practitioners on the use of realist evaluation to assess programmes and policies in LMICs. In doing so, we also situate our work within broader conversations on knowledge production and positionality, including insights from the closing plenary on ‘Whose knowledge counts?’ and our organized session on realist evaluations at HSR 2024. By critically reflecting on the process of planning, conducting, and disseminating these evaluations, we underscore the importance of reflexive practice among realist evaluation practitioners, particularly in navigating epistemic tensions, power asymmetries, and contextual complexity (Abimbola 2021). Throughout the paper, we return to the idea that realist evaluation is a versatile toolkit rather than a guarantee: it can support more equitable knowledge production when its principles—attention to mechanisms, context, and outcomes; iterative theory refinement; and engagement with diverse perspectives—are applied thoughtfully and reflexively.

## Implications of realist evaluation for epistemic justice in health policy and systems research

**Figure 1.**
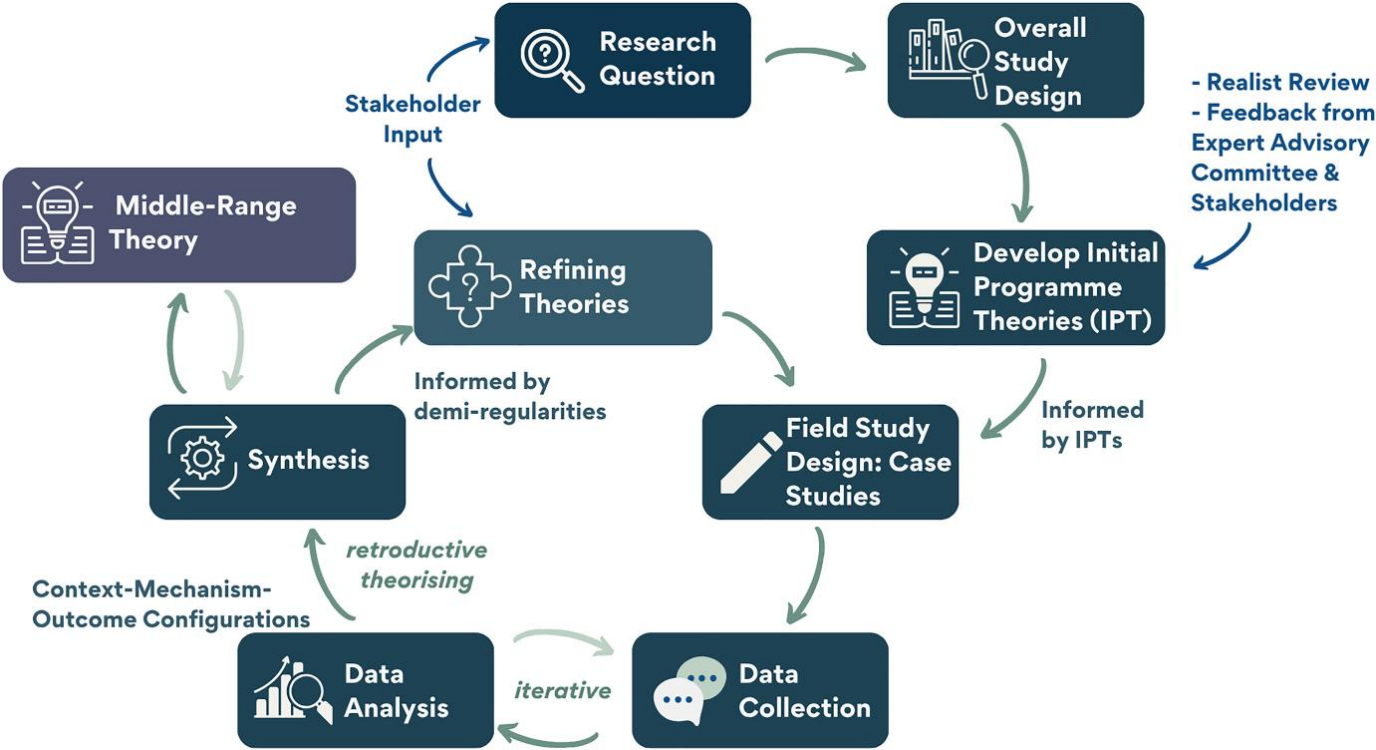
The realist evaluation cycle, adapted from Dada et al. (2025).

The logic of realist evaluation is that researchers can move beyond simple effectiveness analyses to develop in-depth explanations of how policies, programmes, and interventions generate outcomes through contextually contingent mechanisms. Supplementary Box S1 presents several of the key terms and concepts in realist research. Realist studies are anchored in an iterative, theory-driven approach that seeks to elucidate how and why interventions function within specific contexts, emphasizing the dynamic interplay through context-mechanism-outcome configurations (CMOCs). This iterative evaluation approach (Fig. 1) allows researchers, policymakers, practitioners, and communities to generate (and contribute, transparently and equitably to generating) actionable insights that enhance the design and implementation of health policies, programmes, and interventions in diverse settings. It does so by creating the space at each stage of the realist evaluation cycle for such engagement in generating and refining the theories used to explain their outcomes. After all, given their inherent complexity, social policies, programmes, and interventions are influenced by—and in turn influence—the socio-political structures that shape their implementation and impact (Hewitt et al. 2012, Graham and McAleer 2018, Nielsen et al. 2022). The evaluation of social policies and programmes should incorporate considerations of such complexity of causation and the power dynamics that shape their implementation and impact and power dynamics among researchers or between researchers and other actors which may influence their evaluation.

## Theoretical and practical insights from conducting realist evaluations

Applying realist evaluation presents both theoretical and practical challenges, requiring researchers to navigate issues of contextual complexity, power dynamics, and knowledge translation. This section reflects on our experiences conducting two realist evaluations, highlighting key challenges and strategies in designing, conducting, and translating realist research into policy and practice.

### Deciding to use a realist evaluation

Our decisions to use realist evaluation stemmed from the need to move beyond ‘what works’ to understand ‘what works, for whom, in what circumstances, and why’ (Pawson and Tilley 1997a, 1997b). This required engaging with a broad range of actors who possess knowledge of the setting—not only by virtue of formal roles but also through their lived experience, positionality, or informal influence within the system. Such actors are often excluded in conventional research approaches, where methodological boundaries or funding priorities tend to privilege elite or easily accessible voices. Given that both intersectoral collaboration and community engagement operate in multi-layered environments, we needed an approach that could

- Unpack the mechanisms underlying power and communication: in intersectoral collaboration, power dynamics between programme managers, bureaucrats, and frontline implementers shape policy coordination and resource allocation (Mondal et al. 2021). Similarly, communication plays a significant role in community engagement, influenced by how community health volunteers, local leaders, and community members interact and relate to each other (Van Belle et al. 2017).
- Account for context-specific variations: the same intervention may produce different outcomes depending on political will, institutional structures, local leadership, and socio-cultural factors. Realist evaluation allows for comparative insights across contexts (Nielsen et al. 2022).
- Capture the iterative and adaptive nature of health system policies, programmes, and interventions: unlike experimental designs that assume stability, realist evaluation accommodates continuous learning and adaptation, making it well-suited for examining how intersectoral collaboration and community engagement evolve over time and how this changes the contexts/environments where they function (Lloyd 2018, Nielsen et al. 2022).
- Bridge research with policy and practice: by developing empirically grounded CMOCs, realist evaluation produces explanatory insights that inform programme design and implementation. For instance, identifying how trust between sectors enables shared decision-making can help tailor coordination strategies to improve collaboration in specific settings (Punton and Vogel 2020).

In both studies, our choice of realist evaluation was not only methodological but also epistemological—an effort to shift from dominant, externally driven models of evaluation to approaches that are grounded in local realities and shaped by diverse actors. This shift allowed us to engage more deeply with the knowledge and interpretations of people directly involved in policy implementation and community action, ensuring that their perspectives informed not just the findings but also the framing of the research questions. In doing so, we aimed to advance epistemic justice by attending to the distributed nature of knowledge in intersectoral collaboration and community engagement and by contributing to the evolving theory and practice of context-sensitive and explanatory HPSR.

### Planning a realist evaluation

Planning a realist evaluation involves careful balancing of theoretical rigour and practical feasibility (Punton et al. 2016). However, operationalizing this planning phase, including the development of data collection approaches, is often not described in detail in published realist evaluations.

An initial consideration in planning a realist evaluation involves putting together a research team. Unlike traditional evaluations that prioritize measuring outcomes and progress against predefined indicators, realist evaluation demands a nuanced understanding of CMOCs and a theory-driven, iterative approach to inquiry. This shift calls for a team that blends methodological flexibility with critical thinking and is open to grappling with complexity and abstraction. In practice, this means selecting team members not only for their evaluation experience but also for their reflexivity, interdisciplinary orientation, and ability to engage diverse programme, policy, or intervention actors in theory elicitation and testing. In the Zambia study, the lead researcher worked closely with a local research assistant to invest early in joint learning—through data workshops, co-learning sessions, and close mentoring—to develop a shared understanding of realist principles and language.

Importantly, the approach, methods, and case selection in a realist evaluation are guided by the PTs being tested (Manzano 2016). Selecting an appropriate case study for a realist evaluation facilitates the testing and refinement of PTs by examining how the programme works in diverse contexts through different mechanisms (Pawson and Tilley 1997a, 1997b). After identifying the case (an ongoing community engagement initiative in Zambia, the Safe Motherhood Action Groups), the researcher adapted theories first developed in a realist synthesis (Dada et al. 2023) to the project setting through discussions with local project stakeholders and observations of the programme to reflect best the realities of the programme being evaluated (Dada et al. 2025).

Similarly, in the India evaluation, PTs were co-developed through document analysis, stakeholder interviews, and an exploratory qualitative study (Aivalli et al. 2024). By centring the voices of frontline implementers and surfacing tacit knowledge often excluded from policy discourse and evaluation, this early phase avoided enhanced epistemic justice. Using a reflexive realist approach, researchers engaged intersectoral actors to examine power differentials in how knowledge is generated and valued. The PTs were later refined through a realist synthesis Aivalli et al., submitted for publication and used to guide the selection of a district implementing a nutrition mission, ensuring contextual and political relevance for the realist evaluation.

Researchers also considered the types of evidence—in both cases, primarily qualitative—that would best support testing and refining the PTs. While realist interviews often adopt a ‘teacher–learner’ style where theories are presented for participant reflection, both evaluations also identified ways to move beyond this (described further in the next section). Once qualitative data collection was determined as the primary data collection technique, a key challenge in communicating with participants was balancing the conceptual depth of PTs with accessibility for participants. Concepts such as ‘mechanism’ and ‘outcome’ were often abstract to participants, requiring rephrasing into relatable language. Relatedly, conducting debriefs within the research team in Zambia after each interview helped identify these challenges, helping to refine questioning and adjust phrasing for future interviews (Dada 2025, Dada et al. 2025). This aligns with other realist researchers’ experiences, highlighting iterative team reflection as key to improving data quality and interview responsiveness (Gilmore 2019, Hebbar et al. 2022). This iterative adaptation helped achieve greater clarity and deeper engagement with emerging theories. For example, when participants in Zambia simply agreed with a PT without elaboration, researchers shifted the strategy by focusing on components of the theory—for instance, asking ‘what makes you trust someone?’ rather than presenting the entire PT on trust. Such adaptations were instrumental in eliciting richer data. These planning processes facilitated the more equitable construction of PTs, particularly by elevating insights from actors often marginalized in formal policymaking. In doing so, they contributed to addressing knowledge (and knower) hierarchies—a central concern in advancing epistemic justice within HPSR.

### Engaging stakeholders in data collection and analysis

Realist evaluation in HPSR often benefits from engaging with diverse actors, including policymakers, programme implementers, frontline health workers, and community representatives. However, engaging with these actors can present challenges relating to power and hierarchy. In India, government actors often operated within rigid and hierarchical bureaucratic systems, making intersectoral engagement difficult and often constrained. Eliciting candid reflections across sectors required navigating these institutional hierarchies with care and strategic sensitivity, as evidenced in two independent realist studies (Aivalli, Hebbar et al. 2025). Junior officials were frequently reluctant to discuss structural or institutional constraints openly, often due to concerns about potential repercussions. In contrast, senior officials tended to frame their responses aspirationally, often omitting operational challenges. To elicit more candid and grounded insights, the researcher complemented formal interviews with informal interactions, off-the-record conversations, and group discussions, creating safer spaces for reflection and dialogue. These approaches enabled the surfacing of tacit, experiential knowledge, and nuanced accounts that were essential for refining PTs.

The Zambia realist evaluation also faced challenges with power in data collection. As an ‘outsider’ to the community who was affiliated with a high-income-country-based institution, the researcher was aware of the power dynamics at play and wanted to acknowledge these. For example, the researcher explored the use of a participatory method (e.g. photovoice) in the realist evaluation as a way to effectively engage with one of the participant populations (Mukumbang and Dada 2024). The approach to developing and using this participatory tool points to the alignment of realist research with participatory methods, highlighting the opportunity for innovation and customization in the realist evaluation, in ways that give voice to actors whose knowledge and interpretations may otherwise be silenced are not (Dada 2025).

A key challenge in both studies was addressing knowledge hierarchies when synthesizing data across system levels. To promote equitable representation of diverse voices, we undertook iterative, team-based cross-checking of emerging CMOCs. For example, the India study involved collaborative sessions where researchers, field staff, and domain experts reviewed coded data, compared interpretations across hierarchies, and ensured that marginalized perspectives—particularly from frontline and community actors—were not overshadowed by elite narratives. Particular attention was given to avoiding oversimplification or privileging elite narratives. These approaches—including informal conversations, participatory methods, and team-based analysis—were not only practical responses to field challenges but also intentional strategies to centre voices that are often marginalized in policy evaluations. By creating more comfortable and trusting spaces for conversation, and by involving frontline workers and community members in making sense of the findings, we were able to uncover everyday experiences, small acts of resistance, and local knowledge that are often missed in traditional, top-down evaluations. This aligns with community-embedded realist approaches, where co-production and trust enabled the surfacing of local knowledge often missed in conventional evaluations (Prashanth et al. 2025). This participatory and iterative engagement is conscious of power dynamics and critical to promoting epistemic justice in HPSR, centring a broad range of knowledge, interpretations, and experiences.

### Refining theories iteratively amidst logistical constraints

Operationalizing and sustaining the iterative nature of realist evaluations often presents significant challenges including logistical limitations, institutional fluidity, and access-related constraints. For example, in the India case study, a major barrier to iterative refinement of PTs with stakeholders was the frequent transfer and reassignment of programme managers and administrative personnel, a common feature in Indian bureaucratic systems (La Forgia et al. 2015, Garimella and Sheikh 2016). This resulted in the loss of continuity in relationships and institutional memory, as stakeholders familiar with the study’s objectives and earlier rounds of data collection were often replaced by new officials. To mitigate this, the researcher broadened its respondent base to include actors across multiple administrative tiers and departments, thereby ensuring that theory development was not overly reliant on any single individual or role. Building relationships with mid-level functionaries and frontline staff, who were often more stable in their postings, also proved critical for maintaining continuity. Another significant challenge was the limited availability and competing demands on stakeholders’ time. Government officials and programme implementers were often operating under intense workloads, particularly in settings where multiple programmes were being implemented concurrently. Many participants had only brief windows for engagement, necessitating a flexible and adaptive approach. Interviews were often conducted in informal settings, during field visits, or alongside programme review meetings to maximize opportunities for dialogue. Follow-up conversations were scheduled opportunistically, sometimes requiring multiple shorter engagements rather than a single extended interview.

The iterative nature of realist work also required researchers to revisit emerging PTs with participants for feedback and validation. Yet, under such logistical constraints, traditional, formalized approaches were not always feasible. Instead, the researcher in the India study adopted informal validation strategies, including summary debriefings with key informants, peer validation, and triangulation with documentary evidence and prior observations. These adaptations allowed for the gradual sharpening of CMOCs without placing undue burden on participants already constrained by time and administrative responsibilities. Embracing an adaptive and relational approach allowed the evaluation to remain theoretically robust while responsive to contextual realities. Flexibility and creative problem-solving are thus essential in realist evaluation, particularly in settings with resource constraints or hierarchical systems where access and continuity cannot be taken for granted.

The iterative refinement of theories, despite institutional instability and limited access to decision-makers, highlighted the importance of distributed and collective knowledge in health systems. By diversifying our respondent base and relying on mid-level and frontline actors, we moved closer to an epistemically just approach, where theory building is not monopolized by a few powerful voices but informed by actors embedded in daily practice. This highlights how realist evaluation can fulfil the need for methodological flexibility in HPSR to accommodate political and relational realities.

### Disseminating and translating findings into policy and practice

A persistent challenge in realist evaluation lies in ensuring that its findings—while theoretically rich and contextually grounded—are translated into actionable and accessible insights for policymakers, programme implementers, and practitioners. The complexity of CMOCs and theories, though central to explanatory depth, can be difficult to communicate without oversimplification (Aivalli 2025). Navigating this tension required a deliberate, multi-pronged dissemination strategy adapted to each setting. In India, dissemination efforts focused on translating findings into practical insights for district and block administrators, and frontline programme implementation staff. While formal briefs were not produced, findings were shared through targeted presentations and informal dialogues with intersectoral actors from the Departments of Health, Social Welfare, and Education. These engagements were tailored to highlight how the identified mechanisms—such as trust building, clarity of roles, and informal leadership—could be harnessed to improve routine intersectoral coordination. In some cases, informal debriefs with field-level implementers served both as a form of member validation and as an avenue for knowledge translation. In Zambia, the researcher met and discussed the findings with actors (at the district, provincial and national levels of the Ministry of Health), implementation partners, non-profits, and the communities where the evaluation was conducted and shared summaries of the findings with these groups in the form of written briefs. Local partners also helped to identify key working groups and avenues where to present findings in order to inform future policies and programming. Creative dissemination approaches (e.g. animated videos) were also used to provide more visual and accessible forms of communication and were initially shared with the same groups of stakeholders where possible.

Given the common elements of both the studies, we also reflected on whose voices were amplified in the research translation process. In many LMIC settings, evidence used in policymaking remains dominated by external institutions or outside researchers. Ensuring that knowledge generated through realist evaluation was locally owned and used required strong partnerships with government actors, community organizations, and frontline implementers throughout all the stages involved in the realist evaluations. The examples described above aimed to contribute to addressing this challenge and ensuring the findings of the realist evaluations could have practical and pragmatic impact.

## Conclusion

Realist evaluation offers a powerful lens for unpacking complexity in health systems, particularly in understanding how context and mechanisms interact to shape outcomes. As demonstrated through our examples in India and Zambia, its application goes beyond analytical rigour—it enables researchers to engage with questions of power, positionality, and justice in both the conduct and use of research. However, the potential of realist evaluation to advance equity and epistemic justice remains constrained by persistent structural challenges, including limited capacity to undertake realist evaluation in LMICs, the concentration of training opportunities in high-income countries, and barriers to embedding realist insights in policy processes. Addressing these challenges will require intentional and sustained investment in building local realist capacity globally. At present, most formal realist training opportunities are hosted by institutions in high-income countries and are financially inaccessible for many researchers in LMICs. This underscores the importance of supporting and expanding regionally grounded, low-cost training initiatives, such as the Indian Realist Learning Network (Institute of Public Health Bengaluru 2024) and its training programmes, to promote equitable knowledge exchange and capacity building in realist evaluation. Expanding such platforms is essential for enabling diverse researchers to lead context-sensitive, theory-driven evaluations that reflect lived realities and challenge extractive forms of evidence production, contributing to justice-driven research that shapes equitable and sustainable health systems.

## Supplementary Material

czaf080_Supplementary_Data

## Data Availability

No specific data informed this manuscript; however, data from the original project is available upon request.

## Reflexivity statement

The authorship team brings together diverse perspectives across gender, seniority, disciplinary background, and geographic location. The group includes three female and three male authors, ranging from early career to senior researchers, working across institutions in Ireland, India, Nigeria, and Australia. Collectively, the authors have expertise in health systems, health policy, public health, and realist methodology, with significant experience in both high-income and LMIC contexts. Several authors are based in and actively engaged with HPSR in Africa and South Asia, while others contribute perspectives from Europe and Australia, enabling a balance between global and local insights. This diversity of positionality and experience informed both the conceptual framing and the critical reflections presented in this paper.
