# Supplementary material for: How to do (or not to do) realist evaluations to advance theory, practice, and justice in health systems research
Source: Health Policy Plan. 2026 Jun 29;41(Suppl 1):i83–90. doi: 10.1093/heapol/czaf080 (PMC13311662; doi:10.1093/heapol/czaf080)
Supplement: czaf080_Supplementary_Data [file czaf080_supplementary_data.zip › Supplimentary file 1.docx]

**Box 1**

| **Box 1: Key realist terms and concepts** |
| --- |
| **Context-Mechanism-Outcome configuration (CMOC)** – CMOCs are an analytical tool to explain generative causation, that is how particular mechanisms are triggered in specific contexts to produce intended or unintended outcomes (Pawson & Tilley, 1997).  **Context -** The features or conditions of a setting where a program is introduced that influences whether or not mechanisms operate (Pawson & Tilley, 1997; Pawson & Tilley, 2004).  **Mechanism** - The generative forces that drive change (Lacouture *et al.*, 2015). These underlying causal forces are often broken down as ‘resources’ and ‘reasoning.’ The ‘resource’ is what is potentially introduced or provided and the ‘reasoning’ is the response or what prompted changes in behaviour which generates the outcome (Pawson & Tilley, 2004; Dalkin *et al.*, 2015). These mechanisms do not function in isolation but are embedded within particular social, organisational, and political contexts that shape their activation and effectiveness (Dalkin *et al.*, 2015).  **Outcome** – The final, intended or unintended, effects generated by a mechanism in a particular context (Pawson & Tilley, 1997; Pawson & Tilley, 2004).  **Demi-regularities** - Patterns or tendencies in social systems that, while not universally deterministic, exhibit recurring regularities under similar contextual conditions (Ragin & Byrne, 2009; Pawson, 2013).  **Initial Program Theories (IPTs)** - Provisional hypotheses constructed through a synthesis of existing literature, stakeholder expertise, and/or preliminary empirical observations that propose plausible explanations for how interventions might trigger change (Pawson & Tilley, 1997; Greenhalgh *et al.*, 2017).  **Program Theories (PTs)** – IPTs may be refined into PTs through empirical inquiry where researchers examine their applicability in real-world scenarios (Dalkin *et al.*, 2015).  **Middle-Range Theories (MRTs)** - Middle-range theories serve as an ‘intermediate theory’ between the day-to-day working hypotheses and the unified theories that explain social phenomena (Merton, 1968). In realist research, middle-range theories are at a higher level of abstraction than program theories; middle-range theories are specific enough to explain the examined intervention but general enough to be applied in other cases (Pawson, 2000; Wong *et al.*, 2013; Robert *et al.*, 2017).  **Retroduction** - Causal explanations are developed through a process known as retroduction, which involves identifying and refining underlying causal mechanisms through iterative cycles of theory-building and empirical testing (Wong *et al.*, 2017). Retroduction integrates inductive reasoning (deriving theories from data), deductive reasoning (testing theories against existing evidence), and abductive reasoning (generating creative inferences to refine theories) (Pawson & Manzano-Santaella, 2012; Jagosh, 2020). |

References

Dalkin S.M. *et al* (2015). ‘What’s in a mechanism? Development of a key concept in realist evaluation’. *Implementation Science* 10(1), pp.49.

Dalkin S.M. *et al* (2015). ‘What’s in a mechanism? Development of a key concept in realist evaluation’. *Implementation science* 10, pp.1-7.

Greenhalgh T. *et al* (2017). ‘Beyond adoption: a new framework for theorizing and evaluating nonadoption, abandonment, and challenges to the scale-up, spread, and sustainability of health and care technologies’. *Journal of medical Internet research* 19(11), pp.e8775.

Jagosh J. (2020). ‘Retroductive theorizing in Pawson and Tilley's applied scientific realism’. *Journal of Critical Realism* 19(2), pp.121-130.

Lacouture A. *et al* (2015). ‘The concept of mechanism from a realist approach: a scoping review to facilitate its operationalization in public health program evaluation’. *Implementation science* 10, pp.1-10.

Merton R.K. (1968). *On sociological theories of the middle range [1949]*. na

Pawson R. (2000). ‘Middle-range realism’. *European Journal of Sociology/Archives Européennes de Sociologie* 41(2), pp.283-325.

Pawson R. (2013). ‘The science of evaluation: a realist manifesto’.

Pawson R. & Manzano-Santaella A. (2012). ‘A realist diagnostic workshop’. *Evaluation* 18(2), pp.176-191.

Pawson R. & Tilley N. (1997). ‘An introduction to scientific realist evaluation’. *Evaluation for the 21st century: A handbook* 1997, pp.405-418.

Pawson R. & Tilley N. (1997). *An introduction to scientific realist evaluation*. 1997

Pawson R. & Tilley N. (1997). *Realistic evaluation*. sage

Pawson R. & Tilley N. (2004). ‘Realist evaluation: The magenta book guidance notes’. *London: Cabinet Office. Retrieved May* 12, pp.2008.

Ragin C.C. & Byrne D. (2009). *The Sage handbook of case-based methods*.

Robert E. *et al* (2017). ‘Building a middle-range theory of free public healthcare seeking in sub-Saharan Africa: a realist review’. *Health Policy and Planning* 32(7), pp.1002-1014.

Wong G. *et al* (2013). ‘RAMESES publication standards: realist syntheses’. *BMC Medicine* 11(1), pp.21.

Wong G. *et al* (2017) Health Services and Delivery Research. In *Quality and reporting standards, resources, training materials and information for realist evaluation: the RAMESES II project*, NIHR Journals Library

Copyright © Queen’s Printer and Controller of HMSO 2017. This work was produced by Wong et al. under the terms of a commissioning contract issued by the Secretary of State for Health. This issue may be freely reproduced for the purposes of private research and study and extracts (or indeed, the full report) may be included in professional journals provided that suitable acknowledgement is made and the reproduction is not associated with any form of advertising. Applications for commercial reproduction should be addressed to: NIHR Journals Library, National Institute for Health Research, Evaluation, Trials and Studies Coordinating Centre, Alpha House, University of Southampton Science Park, Southampton SO16 7NS, UK., Southampton (UK).
