# Supplementary figures and images for: How to do (or not to do) realist evaluations to advance theory, practice, and justice in health systems research
Source: Health Policy Plan. 2026 Jun 29;41(Suppl 1):i83–90. doi: 10.1093/heapol/czaf080 (PMC13311662; doi:10.1093/heapol/czaf080)

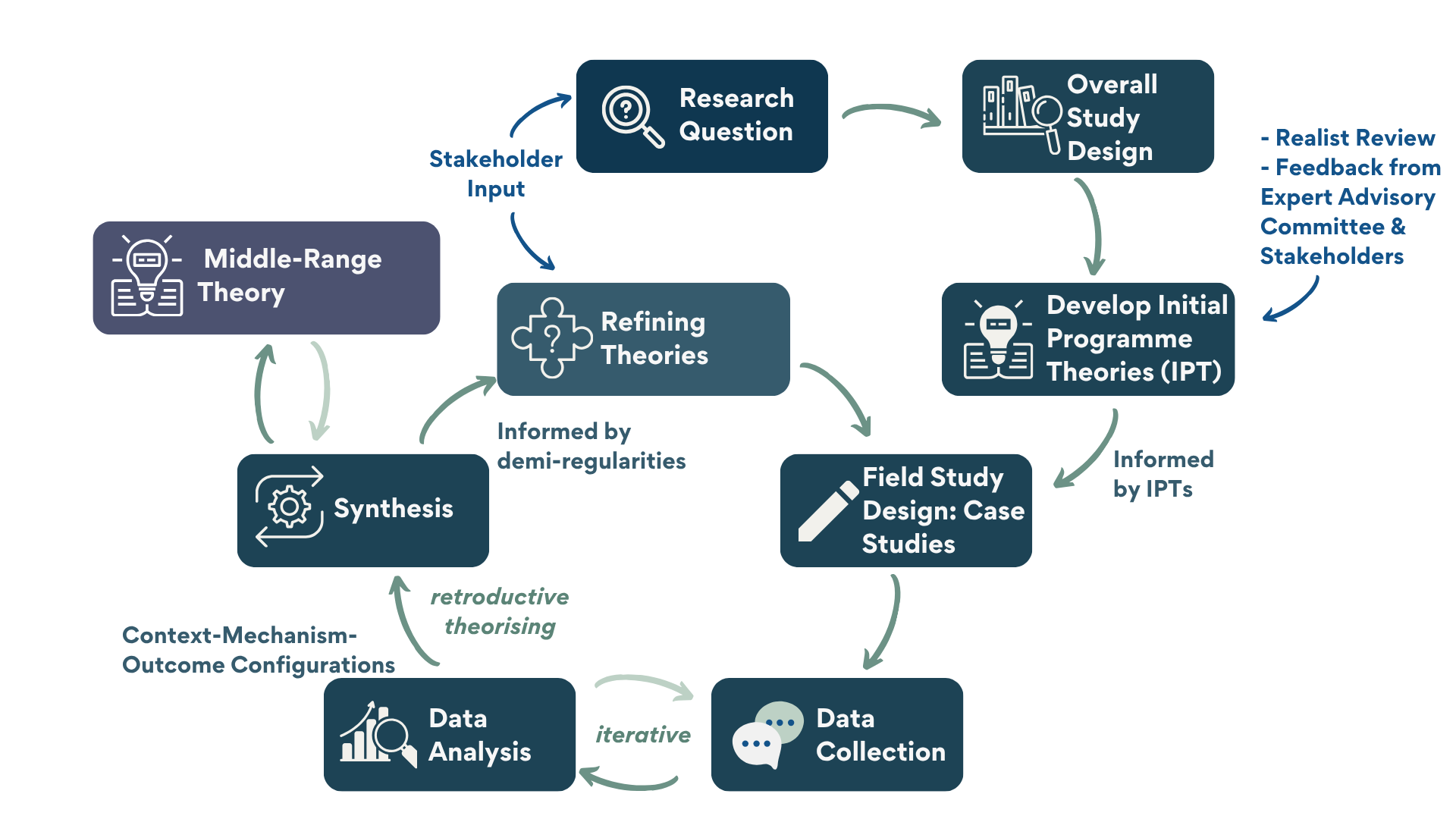

Supplement: czaf080_Supplementary_Data [file czaf080_supplementary_data.zip › Figure 1.tiff]
